# Steric shielding *vs.* σ–π orbital interactions in triplet–triplet energy transfer†
†Electronic supplementary information (ESI) available. See DOI: 10.1039/c5sc00823a


**DOI:** 10.1039/c5sc00823a

**Published:** 2015-05-01

**Authors:** Inmaculada Andreu, Isabel Morera, Fabrizio Palumbo, German Sastre, Francisco Bosca, Miguel A. Miranda

**Affiliations:** a Unidad Mixta de Investigación IIS La Fe–UPV , Hospital La Fe , Bulevar Sur s/n , 46026 Valencia , Spain; b Departamento de Química UPV , Universitat Politècnica de València , Camino de Vera s/n , 46022 Valencia , Spain; c Instituto Universitario Mixto de Tecnología Química (UPV-CSIC) , Universitat Politècnica de València , Avenida de los Naranjos s/n , 46022 Valencia , Spain . Email: fbosca@itq.upv.es ; Email: mmiranda@qim.upv.es

## Abstract

The influence of non-covalent σ–π orbital interactions on triplet–triplet energy transfer (TTET) through tuning of the donor excitation energy remains basically unexplored. In the present work, we have investigated intermolecular TTET using donor moieties covalently linked to a rigid cholesterol (Ch) scaffold. For this purpose, diaryl ketones of π,π* electronic configuration tethered to α- or β-Ch were prepared from tiaprofenic acid (TPA) and suprofen (SUP). The obtained systems TPA-α-Ch, TPA-β-Ch, SUP-α-Ch and SUP-β-Ch were submitted to photophysical studies (laser flash photolysis and phosphorescence), in order to delineate the influence of steric shielding and σ–π orbital interactions on the rate of TTET to a series of energy acceptors. As a matter of fact, fine tuning of the donor triplet energy significantly modifies the rate constants of TTET in the absence of diffusion control. The experimental results are rationalized by means of theoretical calculations using first principles methods based on DFT as well as molecular dynamics.

## Abstract 2

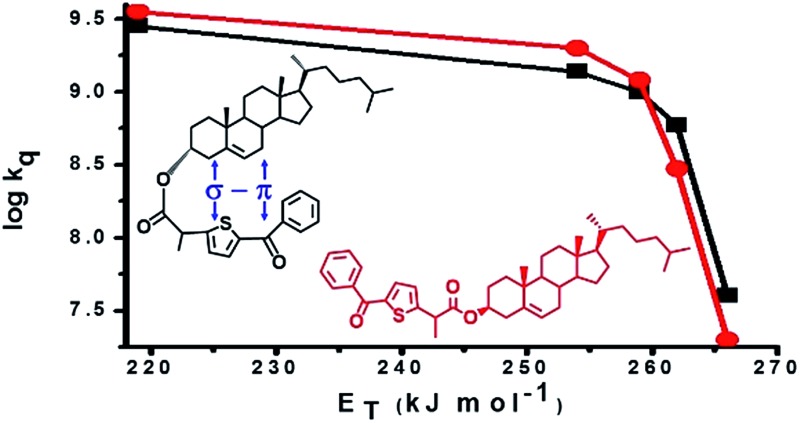
Fine tuning of the benzoylthiophene triplet level through σ–π orbital interactions modifies the energy transfer rate constants to appropriate acceptors.

## Introduction

**Chart 1:**
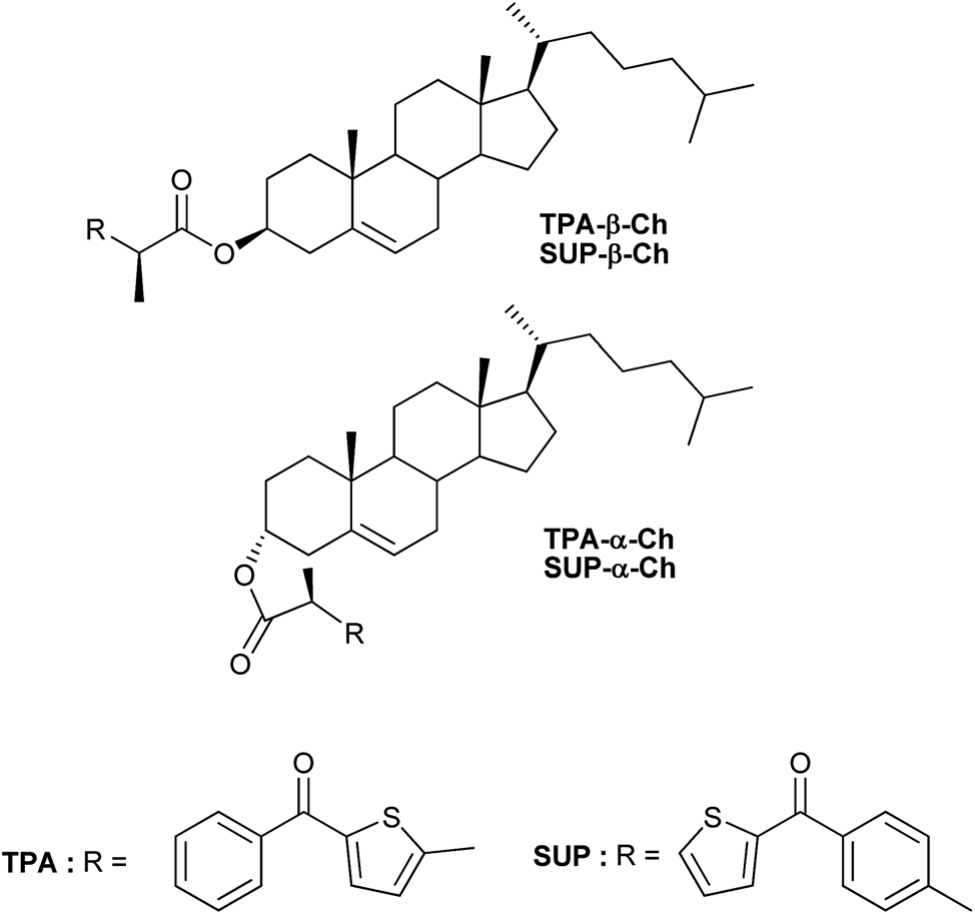
Chemical structures of the investigated compounds.

Triplet excitation energy can be transferred between two chromophores by the Dexter mechanism, which is based on an electron exchange through orbital overlap of the donor excited state and the acceptor ground state.^1^ The rate constants of diffusion-controlled triplet–triplet energy transfer (TTET) are affected by steric hindrance (shielding) as demonstrated using aromatic ketones as donors;^2^ however, the influence of non-covalent σ–π orbital interactions on TTET through tuning of the donor excitation energy remains basically unexplored. Intramolecular TTET by means of through-bond electronic coupling occurs when orbitals of the donor and acceptor chromophores mix slightly with orbitals of an intervening molecular skeleton.^3^,^4^ Based on this concept, it appeared interesting to investigate intermolecular TTET using donor moieties covalently linked to a rigid steroidal scaffold. For this purpose, model systems containing a diaryl ketone of π,π* electronic configuration (to minimize chemical reactivity) tethered to a rigid cholesterol (Ch) scaffold were considered as most appropriate. Accordingly, starting from tiaprofenic acid (TPA) and suprofen (SUP), TPA-α-Ch, TPA-β-Ch, SUP-α-Ch and SUP-β-Ch (Chart 1) were prepared and submitted to photophysical studies, in order to delineate the influence of steric shielding and σ–π orbital interactions on the rate of TTET to a series of energy acceptors. In this context, it was anticipated that only the folded conformation of α-derivatives would allow a close approach between the two linked substructures.^5^–^7^ The experimental results provided a clear proof of the concept, and theoretical calculations (DFT and molecular dynamics) served to rationalize the observed trends.

## Results and discussion

### Photophysical studies

In a first stage, laser flash photolysis (LFP) of TPA-α-Ch and TPA-β-Ch was performed in dichloromethane upon 355 nm excitation (5 mJ pulse^–1^). The transient absorption spectra showed in both cases two broad bands (with *λ*_max_ around 360 and 600 nm, and relative intensities of *ca.* 2 : 1) ascribed to the TPA triplet excited state.^6^

**Fig. 1:**
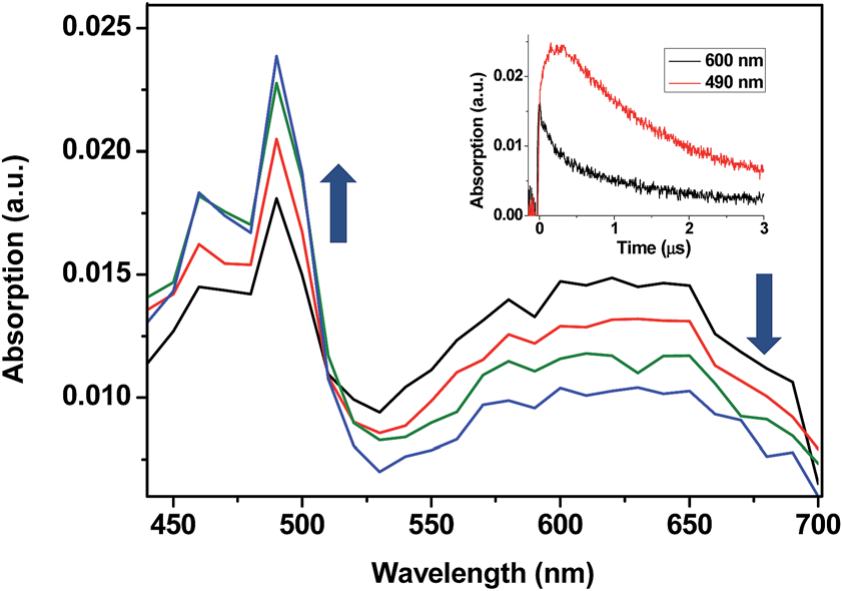
Transient absorption spectra obtained upon 355 nm excitation of TPA-α-Ch (0.1 mM) in N_2_-purged dichloromethane solutions containing PH (3 mM) at 15, 50, 125, and 200 ns after the laser pulse. Inset shows the growth and decay profiles monitored at 490 and 600 nm, respectively.

To determine the energy transfer reactivity, the decay of these transients was examined in the presence of a variety of potential acceptors. The triplet energy (*E*_T_) of TPA is thought to be slightly below that of 2-benzoylthiophene, which has been estimated at 264 kJ mol^–1^.^8^,^9^ Accordingly, the acceptors selected for the study were 4-biphenylcarboxylic acid (BPC), phenanthrene (PH), naproxen (NP), naphthalene (NPH) and 1,3-cyclohexadiene (CHD), whose reported *E*_T_ values are 265,^10^ 262,^11^ 259,^12^ 255 13and 214 14kJ mol^–1^, respectively. Thus, LFP of TPA-α-Ch and TPA-β-Ch was performed in the presence of increasing amounts of BPC, PH, NP, NPH, and CHD, and the decay traces were analyzed at 600 nm in order to determine the quenching rate constants (*k*_q_). In parallel, the growth of the triplet bands corresponding to the acceptors was observed for PH (*λ*_max_ at 490 nm, see Fig. 1),^15^ NP (*λ*_max_ at 430 nm),^10^,^12^ and NPH (*λ*_max_ at 415 nm),^15^ and in the case of BPC energy transfer was too slow. By contrast, the process was diffusion controlled for CHD, but the transient absorption was out of the detection range.^15^

**Fig. 2:**
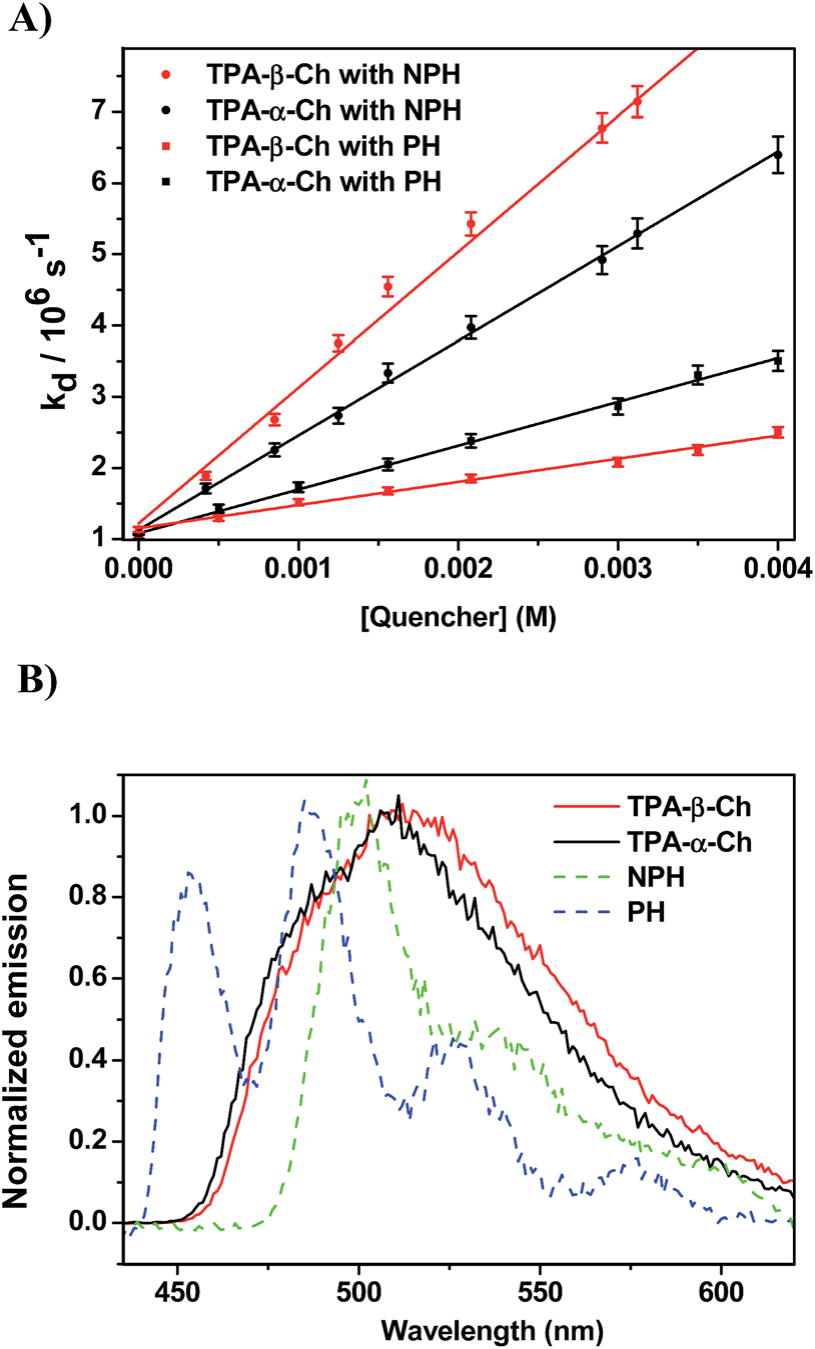
(A) Plot of the triplet decay rate constants (*k*_d_) of TPA-α-Ch and TPA-β-Ch *versus* different concentrations of NPH and PH as quenchers. (B) Normalized phosphorescence emission spectra of TPA derivatives (TPA-β-Ch and TPA-α-Ch), NPH and PH in dichloromethane at 77 K.

When the decay rate constants (*k*_d_) were plotted against the concentrations, straight lines were obtained, and the *k*_q_ values were determined from the slopes (see Table 1 and Fig. 2A for NPH and PH). Similar information was obtained from the growth profile of the acceptors at the corresponding triplet absorption maxima. For a clarifying analysis of the obtained kinetic data, experimental triplet energies were required for TPA-α-Ch and TPA-β-Ch; the values were determined from phosphorescence spectra (Fig. 2B) and found to be 260 and 258 kJ mol^–1^, respectively.

**Table 1:** Rate constants for intermolecular quenching (*k*_q_) of the triplet states of TPA-α-Ch and TPA-β-Ch by different energy acceptors

Quencher	*E*_T_/kJ mol^–1^	*k*_q (TPA-α-Ch)_/10^9^ M^–1^ s^–1^	*k*_q (TPA-β-Ch)_/10^9^ M^–1^ s^–1^	*k*_q (TPA-α-ChH)_/10^9^ M^–1^ s^–1^
BPC	265^*a*^	0.039 (±0.001)	0.016 (±0.001)	
PH	262^*b*^ (262)^*c*^	0.62 (±0.02)	0.32 (±0.01)	0.58 (±0.03)
NP	259^*d*^ (259)^*c*^	1.04 (±0.01)	1.19 (±0.03)	1.01 (±0.01)
NPH	255^*e*^ (251)^*c*^	1.37 (±0.02)	1.96 (±0.06)	1.41 (±0.03)
CHD	214^*f*^	2.81 (±0.04)	3.66 (±0.03)	

^*a*^Lit. ref. 6.

^*b*^Lit. ref. 7.

^*c*^Determined from the experimental phosphorescence spectra.

^*d*^Lit. ref. 8.

^*e*^Lit. ref. 9.

^*f*^Lit. ref. 10.

**Fig. 3:**
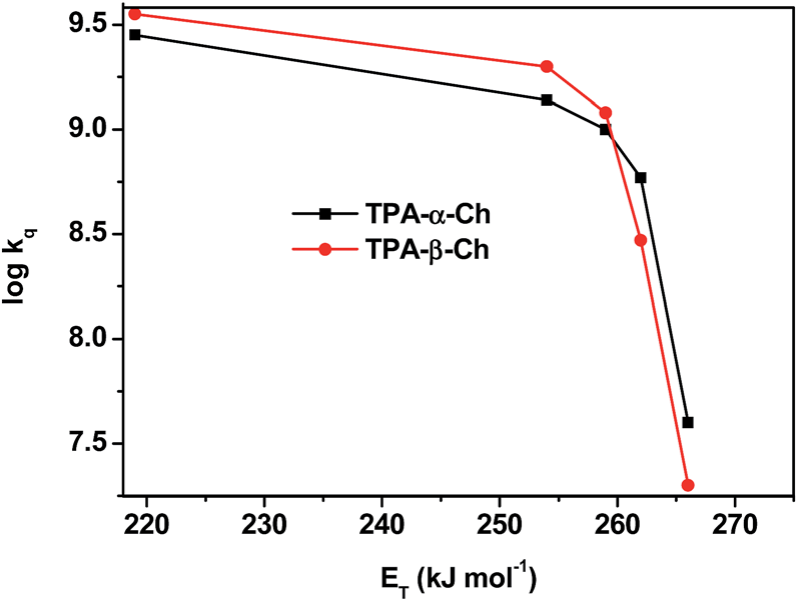
Plot of log *k*_q_ of TPA-α-Ch and TPA-β-Ch *versus* the triplet energy of the acceptors.

The results revealed *k*_q_ values for TPA-α-Ch lower than those obtained for TPA-β-Ch when the process was exothermic and hence close to the diffusion control in dichloromethane (*k*_q_ > 10^9^ M^–1^ s^–1^). This effect should be attributed to the steric shielding effect of cholesterol in TPA-α-Ch, which may adopt a folded conformation. The reverse was true for the case of slightly endothermic processes, indicating that another factor must be playing an important role in this region. Anticipating that it could be associated with the triplet energy differences between TPA-α-Ch and TPA-β-Ch, log *k*_q_ of TPA-β-Ch and TPA-α-Ch was plotted *versus* the triplet energy of quenchers, and a crossing was noticed at *ca.* 259 kJ mol^–1^ (Fig. 3). This would be in agreement with the expectations from Sandros models for vertical endothermic energy transfer,^16^ where the kinetics is basically controlled by the triplet energy gap.

**Fig. 4:**
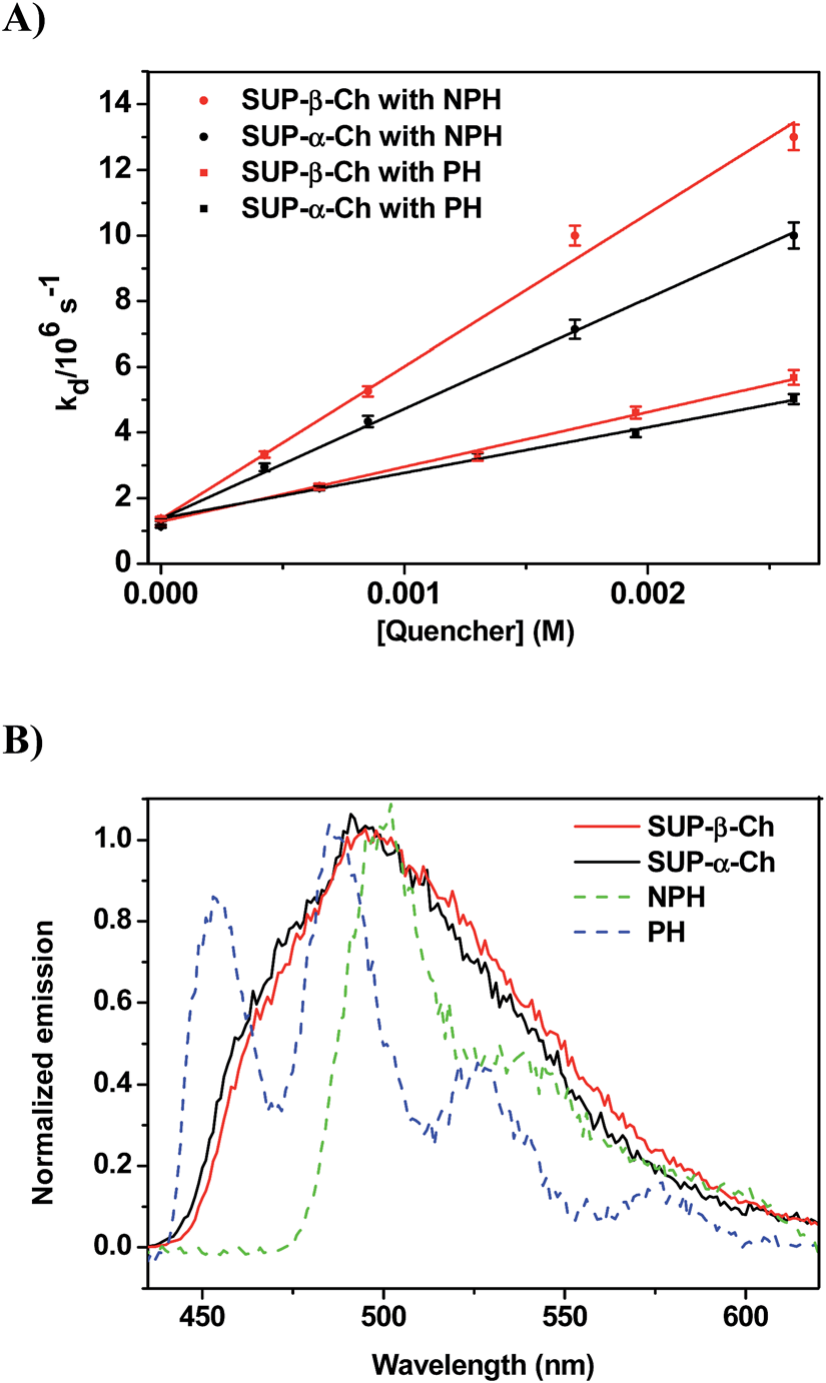
(A) Plot of decay rate constants (*k*_d_) of SUP-α-Ch and SUP-β-Ch (B) *versus* different concentrations of NPH and PH as quenchers. (B) Normalized phosphorescence emission spectra of SUP derivatives (SUP-β-Ch and SUP-α-Ch) and NPH and PH in dichloromethane at 77 K.

In order to confirm the dominating role of the *E*_T_ value of the donor in the less favored region, the studies were extended to SUP-α-Ch and SUP-β-Ch (see results in Fig. 4A). In these systems, the benzoylthiophene chromophore is maintained, but it is attached to the scaffold through the phenyl group. This leaves the thiophene ring unsubstituted, and should result in a slightly higher triplet energy. As a matter of fact, phosphorescence measurements (Fig. 4B) led to 265 and 263 kJ mol^–1^ as triplet energies of the α and β derivatives, respectively.

Again, diffusion-controlled exothermic triplet energy transfer to NPH is sensitive to the steric shielding effect of the Ch scaffold, with *k*_q_ = 3.31 ± 0.09 × 10^9^ and 4.65 ± 0.21 × 10^9^ M^–1^ s^–1^ for SUP-α-Ch and SUP-β-Ch, respectively. Interestingly, the uphill process with PH as acceptor is associated with a smaller energy gap, and therefore the discrimination between SUP-α-Ch and SUP-β-Ch as donors (*k*_q_ = 1.47 ± 0.04 × 10^9^ and 1.67 ± 0.06 × 10^9^ M^–1^ s^–1^ respectively) is much less marked than in the analogous TPA derivatives (compare Fig. 4A and 2A).

**Chart 2:**
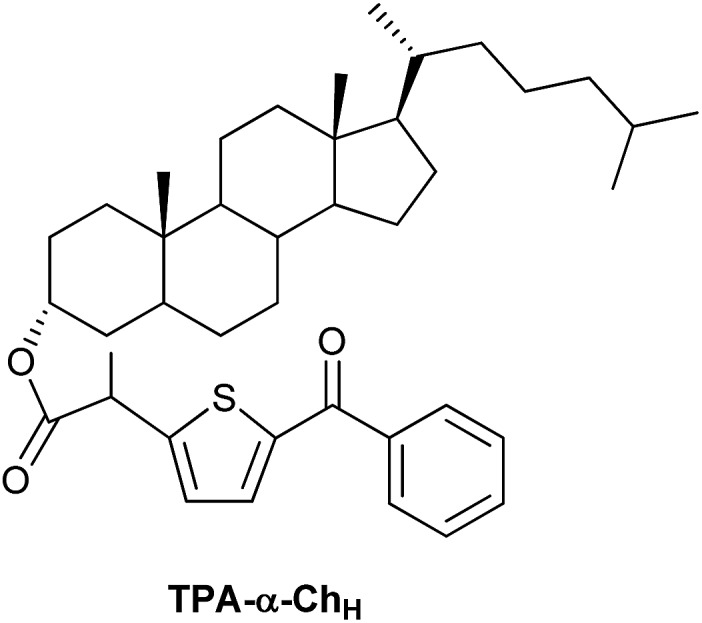
Chemical structure of the TPA derivative of hydrogenated cholesterol.

The non-covalent orbital interactions between Ch and the benzoylthiophene triplet excited state could in principle be of π–π or σ–π nature. In order to evaluate the possible role of π–π interactions in TPA-α-Ch, the double bond was hydrogenated using Pd/C as catalyst, and then the hydroxyl group was esterified with TPA, to give TPA-α-Ch_H_ (see structure in Chart 2).

When LFP experiments were performed with TPA-α-Ch_H_ under the usual conditions, similar photophysical data were obtained as for TPA-α-Ch (see Table 1). Furthermore, the phosphorescence spectrum of TPA-α-Ch_H_ was nearly superimposable to that of TPA-α-Ch. These results allow ruling out a significant influence of π–π orbital interactions on the triplet excited state energy of the benzoylthiophene chromophore when bound to Ch and strongly support the occurrence of σ–π orbital interactions.

### Computational results

Two different types of calculations have been performed with TPA-α-Ch and TPA-β-Ch: minimum energy, using first-principles methods based on DFT (in order to find the most stable conformations) and molecular dynamics, based on an atomistic approach (in order to analyze the conformational dynamics).

**Fig. 5:**
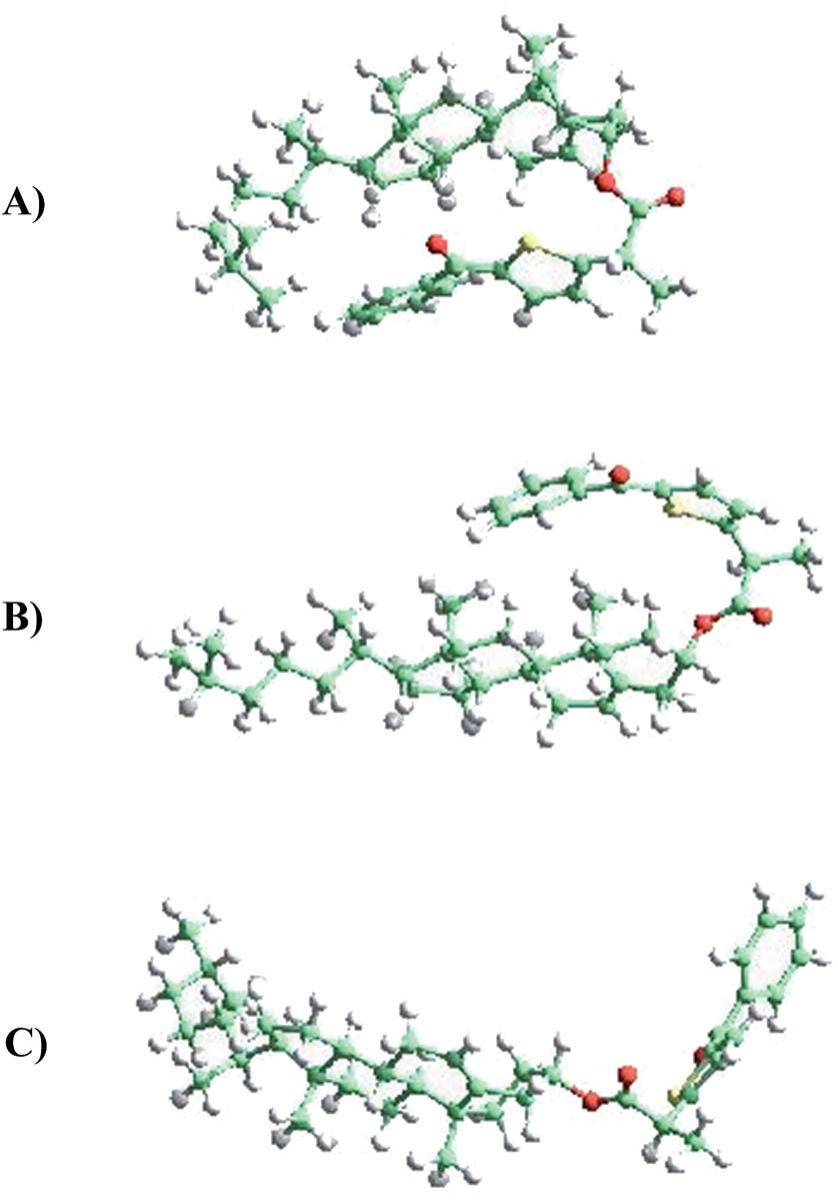
(A) TPA-α-Ch (folded), (B) TPA-β-Ch (folded), (C) TPA-β-Ch (unfolded).

The first-principles DFT energy minimizations showed that for the α-epimer in the triplet excited state, the folded conformation shown in Fig. 5A, is by far the most stable one. In the case of the β-epimer, both the folded and unfolded conformations (Fig. 5B and C) come into play. The geometries of the corresponding conformations in the ground state were almost identical (see ESI†).

The values obtained for the energies of the ground state and triplet excited state of the folded conformations (using three different functionals) are given in Table 2. In general, they were in reasonable agreement with the experimental data and showed the expected trend (triplet energy slightly higher for TPA-α-Ch than for TPA-β-Ch). For instance, with wB79XD, the triplet energy for TPA-β-Ch was 244 kJ mol^–1^, lower than that calculated for TPA-α-Ch (249 kJ mol^–1^). The experimental values are 258 kJ mol^–1^ (TPA-β-Ch) and 260 kJ mol^–1^ (TPA-α-Ch). Thus, the absolute values show a reasonable agreement within 5 kJ mol^–1^. For M062X-D3 a similar semiquantitative agreement was found, with 263 kJ mol^–1^ for the TPA-α-Ch triplet and 263 kJ mol^–1^ for the TPA-β-Ch triplet. Finally, PBE-D3 gave less accurate values, as expected from the usually better performance of M062X and wB97XD for triplet energies. The effect of solvent (dichloromethane) did not have significant influence on the calculations; the calculated energy values in the absence of solvent are given in parenthesis.

**Table 2:** Relative energies (kJ mol^–1^) of minimum energy conformations of ground state (S_0_) and triplet excited state (T_1_) of TPA-α-Ch and TPA-β-Ch in dichloromethane using three different functionals including dispersion: PBE-D3, M062X-D3 and wB97XD. The values in the absence of solvent are given in parenthesis

	PBE-D3	M062X-D3	wB97XD	Exp.
Folded TPA-α-Ch (S_0_)	0 (0)	0 (0)	0 (0)	
Folded TPA-α-Ch (T_1_)	226 (229)^*a*^	263 (267)^*a*^	249 (252)^*a*^	260
Folded TPA-β-Ch (S_0_)	21 (25)^*a*^	26 (31)^*a*^	29 (34)^*a*^	
Folded TPA-β-Ch (T_1_)	216 (214)^*b*^	263 (264)^*b*^	244 (244)^*b*^	258

^*a*^Relative to TPA-α-Ch (S_0_).

^*b*^Relative to TPA-β-Ch (S_0_).

**Fig. 6:**
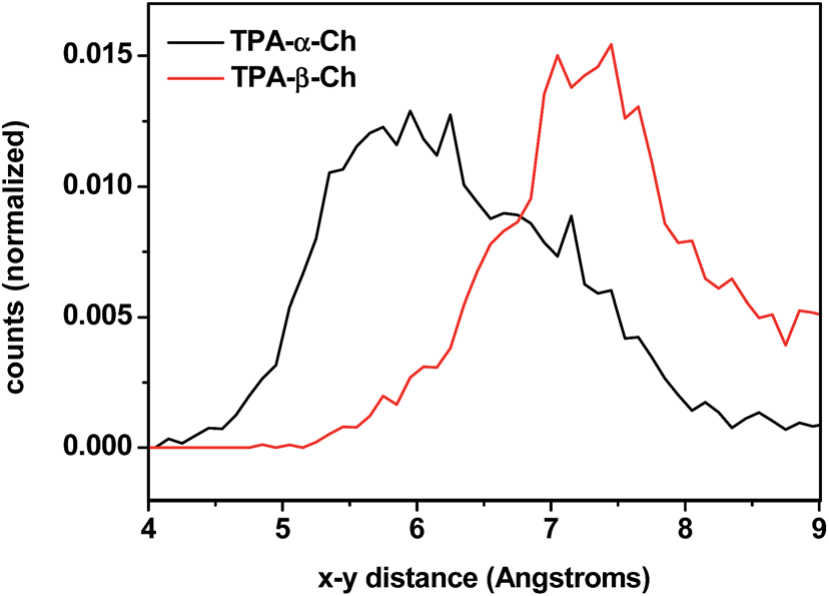
Plot of chromophore–scaffold distances between the carbon of the CO group and the CH of ring B closest to its methyl group found in the molecular dynamics 4 ns runs of TPA-α-Ch and TPA-β-Ch at 298 K in the presence of dichloromethane as solvent.

The results of molecular dynamics simulations are presented in Fig. 6, where the relative populations are plotted *versus* the chromophore–scaffold distance for each conformation in the ground state, up to 9 Å. It is clear that the two moieties are closer to each other in the case of TPA-α-Ch, as the conformations with shorter distance are more heavily populated. This is consistent with the geometries depicted in Fig. 5, where it is apparent that the shape of the α-epimer is more compact.

## Experimental

### General

β-Ch and SUP were commercially available. TPA was soxhlet extracted with dichloromethane from Tiafen®. Commercial solvents and reagents were used without additional purification. ^1^H NMR and ^13^C NMR spectra were recorded in CDCl_3_ as solvent on a Bruker AC-300 at 300 and 75 MHz respectively; NMR chemical shifts are reported in ppm downfield from an internal solvent peak. All reactions were monitored by analytical TLC with silica gel 60 F_254_ revealed with ammonium molybdate reagent. The residues were purified through silica gel 60 (0.063–0.2 mm). Exact mass was obtained by VG Autospec-high-resolution mass and Waters ACQUITY™ XevoQToF spectrometers.

### Synthesis of SUP-α-Ch

To a solution of racemic SUP (375 mg, 1.45 mmol) in CH_2_Cl_2_ (15 mL), dicyclohexylcarbodiimide (DCC, 535 mg, 2.60 mmol) was added portionwise, and the mixture was stirred at 0 °C for 30 min. Then, a solution of α-Ch (500 mg, 1.30 mmol) in CH_2_Cl_2_ (15 mL) and 4-dimethylaminopyridine (DMAP, 20 mg, 0.16 mmol) were added, and the mixture was stirred for further 8 h at the same temperature. Afterwards, the crude reaction was filtered through a pad of Celite®. The resulting filtrate was washed with brine, water, dried over Na_2_SO_4_ and evaporated to dryness under reduced pressure. The obtained residue was purified by column chromatography (eluent: hexane–dichloromethane–ethyl acetate 90 : 5 : 5 v/v/v) to yield a diastereomeric mixture of the corresponding esters. After crystallization from hexane–ethyl acetate (95 : 5 v/v), the (*S*)-SUP-α-Ch diastereoisomer was obtained as a colorless oil (319 mg, 39%) and (*R*)-SUP-α-Ch as a white solid (335 mg, 41%). The latter was used for photophysical studies and labelled as SUP-α-Ch in a simplified way.

#### (*S*)-SUP-α-Ch

^1^H NMR (CDCl_3_, 300 MHz) *δ* 0.58 (s, 3H), 0.80 (d, *J* = 6.6 Hz, 3H), 0.81 (d, *J* = 6.6 Hz, 3H), 0.83 (d, *J* = 6.6 Hz, 3H), 0.89 (s, 3H), 1.47 (d, *J* = 7.2 Hz, 3H), 0.90–1.92 (complex signal, 26H), 2.12 (dm, *J* = 15.3 Hz, 1H), 2.37 (dm, *J* = 15.3 Hz, 1H), 3.71 (q, *J* = 7.2 Hz, 1H), 4.90 (m, 1H), 5.14 (m, 1H), 7.10 (dd, *J* = 4.8 Hz, 3.6 Hz, 1H), 7.36 (dm, *J* = 8.3 Hz, 2H), 7.58 (dd, *J* = 3.6 Hz, 1.1 Hz, 1H), 7.65 (dd, *J* = 4.8 Hz, 1.1 Hz, 1H), 7.77 (dm, *J* = 8.3 Hz, 2H); ^13^C NMR (CDCl_3_, 75 MHz) *δ* 11.8, 18.0, 18.7, 18.9, 20.7, 22.6, 22.8, 23.9, 24.2, 26.0, 28.0, 28.2, 31.8, 32.0, 33.5, 35.8, 36.2, 36.3, 36.9, 39.6, 39.7, 42.3, 46.1, 50.2, 56.1, 56.8, 71.3, 122.3, 127.8, 129.5, 134.0, 134.5, 136.7, 138.2, 143.7, 145.4, 173.1, 187.5. HRMS (EI): *m*/*z* found 628.3947, calculated for C_41_H_56_O_3_S (M^+^˙) 628.3950.

#### (*R*)-SUP-α-Ch

^1^H NMR (CDCl_3_, 300 MHz) *δ* 0.57 (s, 3H), 0.80 (d, *J* = 6.6 Hz, 3H), 0.81 (d, *J* = 6.6 Hz, 3H), 0.83 (d, *J* = 6.6 Hz, 3H), 0.88 (s, 3H), 1.46 (d, *J* = 7.2 Hz, 3H), 0.90–1.94 (complex signal, 26H), 2.09 (dm, *J* = 15.3 Hz, 1H), 2.32 (dm, *J* = 15.3 Hz, 1H), 3.70 (q, *J* = 7.2 Hz, 1H), 4.88 (m, 1H), 4.94 (m, 1H), 7.10 (dd, *J* = 5.1 Hz, 3.9 Hz, 1H), 7.35 (dm, *J* = 8.3 Hz, 2H), 7.59 (dd, *J* = 3.9 Hz, 1.1 Hz, 1H), 7.65 (dd, *J* = 5.1 Hz, 1.1 Hz, 1H), 7.77 (dm, *J* = 8.3 Hz, 2H); ^13^C NMR (CDCl_3_, 75 MHz) *δ* 11.8, 17.8, 18.7, 18.9, 20.8, 22.6, 22.9, 23.9, 24.2, 26.3, 28.1, 28.2, 31.7, 31.9, 33.7, 35.8, 36.1, 36.2, 36.9, 39.6, 39.8, 42.3, 45.9, 50.2, 56.1, 56.8, 71.3, 122.4, 127.8, 129.5, 134.0, 134.5, 136.6, 138.2, 143.7, 145.4, 173.2, 187.4. HRMS (EI): *m*/*z* found 628.3968, calculated for C_41_H_56_O_3_S (M^+^˙) 628.3950.

### Synthesis of SUP-β-Ch

To a solution of SUP (75 mg, 0.29 mmol) in CH_2_Cl_2_ (10 mL), β-Ch (100 mg, 0.26 mmol) in CH_2_Cl_2_ (3 mL) was added dropwise, and the mixture was heated under reflux for 8 h. The reaction mixture was cooled to room temperature and then it was washed with water (3 × 10 mL) and brine (10 mL). The organic phase was dried over Na_2_SO_4_, evaporated and purified by column chromatography (eluent: hexane–dichloromethane–ethyl acetate 90 : 5 : 5 v/v/v) to give the corresponding ester SUP-β-Ch (134 mg, 82%) as a white solid.

^1^H NMR (CDCl_3_, 300 MHz) *δ* 0.69 (s, 3H), 0.87 (d, *J* = 6.6 Hz, 3H), 0.88 (d, *J* = 6.6 Hz, 3H), 0.93 (d, *J* = 6.6 Hz, 3H), 1.01 (s, 3H), 1.55 (d, *J* = 7.2 Hz, 3H), 0.96–2.07 (complex signal, 26H), 2.23 (m, 1H), 2.33 (m, 1H), 3.79 (q, *J* = 7.2 Hz, 1H), 4.62 (m, 1H), 5.37 (m, 1H), 7.18 (dd, *J* = 5.1 Hz, 3.6 Hz, 1H), 7.45 (dm, *J* = 8.1 Hz, 2H), 7.67 (dd, *J* = 3.6 Hz, 1.1 Hz, 1H), 7.73 (dd, *J* = 5.1 Hz, 1.1 Hz, 1H), 7.86 (dm, *J* = 8.1 Hz, 2H); ^13^C NMR (CDCl_3_, 75 MHz) *δ* 11.9, 18.5, 18.7, 19.3, 21.1, 22.6, 22.8, 23.9, 24.3, 27.5, 27.7, 28.0, 28.2, 31.8, 31.9, 35.8, 36.2, 36.4, 36.6, 37.0, 37.8, 38.0, 39.5, 39.7, 42.3, 45.8, 50.0, 56.2, 56.7, 74.6, 122.8, 127.6, 127.9, 129.6, 134.1, 134.7, 136.9, 139.5, 139.6, 143.7, 145.4, 173.3, 187.7. HRMS (EI): *m*/*z* found 628.3933, calculated for C_41_H_56_O_3_S (M^+^˙) 628.3950.

### Synthesis of TPA-α-Ch_H_

This compound was prepared from TPA (100 mg, 0.38 mmol) following the usual procedure (see above), DCC (136 mg, 0.66 mmol), α-Ch_H_ (132 mg, 0.34 mmol) and DMAP (4 mg, 0.033 mmol). After column chromatography (eluent: hexane–dichloromethane–ethyl acetate 90 : 5 : 5 v/v/v), TPA-α-Ch_H_ (167 mg, 78%) was obtained as a white solid.

^1^H NMR (CDCl_3_, 300 MHz) *δ* = 0.64 (s, 3H), 0.76 (d, *J* = 6.5 Hz, 3H), 0.88 (s, 3H), 0.91 (d, *J* = 6.5 Hz, 6H), 1.64 + 1.66 (d + d, *J* = 7.2 Hz, 3H), 0.80–1.99 (complex signal, 31H), 4.06 (q, *J* = 7.2 Hz, 1H), 5.03 (m, 1H), 7.03 (d, *J* = 3 Hz, 1H), 7.47–7.60 (m, 4H), 7.84 (d, *J* = 7.2 Hz, 2H); ^13^C NMR (CDCl_3_, 75 MHz) *δ* = 11.3, 12.0, 18.6, 20.7, 22.5, 22.8, 23.8, 24.1, 26.0, 28.0, 28.2, 31.9, 32.5, 32.8, 32.9, 35.4, 35.7, 36.2, 39.5, 39.9, 41.9, 42.1, 42.5, 54.3, 56.2, 56.4, 71.5, 125.8, 128.3, 129.1, 132.1, 134.6, 138.0, 142.2, 153.2, 153.3, 171.6, 187.7. HRMS (EI): *m*/*z* found 630.4109, calculated for C_41_H_58_O_3_S (M^+^˙) 630.4107.

### Laser flash photolysis experiments

A pulsed Nd:YAG laser was used for excitation at 355 nm. The single pulses were ∼10 ns duration and the energy was from 10 to 1 mJ pulse^–1^. The LFP system consisted of the pulsed laser, the Xe lamp, a monochromator and a photomultiplier made up of a tube, housing and power supply. The output signal from the oscilloscope was transferred to a personal computer. All experiments were performed at room temperature. The samples were dissolved in dichloromethane to have an absorbance *ca.* 0.20 at 355 nm.

### Phosphorescence measurements

Phosphorescence spectra were obtained from a Photon Technology International (PTI, TimeMaster TM-2/2003) spectrofluorometer equipped with a pulsed Xe lamp. The apparatus was operated in time-resolved mode with a delay time of 0.5 ms. Compounds were dissolved in dichloromethane, placed in a quartz tube (5 mm of diameter) and cooled at 77 K. The absorbance of the samples was 0.5 at the excitation wavelength (310 nm). The triplet energies were estimated from the phosphorescence spectra, at the wavelengths corresponding to the 0–0 bands, or (in the case of structureless spectra) to an emission intensity equivalent to 5% of the maximum value.

### Computational methodology

The minimum energy calculations were carried out using first-principles methods based on DFT where three different functionals (PBE-D3 17, M062X-D3 18and wB97XD^19^) were used for the sake of completeness. All these functionals contain corrections^20^ for dispersion terms, which are essential for the correct account of energetics in conformationally large molecules. Calculations have been performed using an updated version of Gaussian 09 software.^21^ For each molecule, the minimum energy conformations of ground and triplet excited states were calculated,^22^ and from these calculations, the triplet energies (energy difference triplet-ground state) were tabulated. Taking into account that the solvent may have some influence on the stability of the ground and the triplet excited states, the calculations were also carried out with solvent, using a continuum solvent model. Molecular dynamics were calculated by means of an atomistic approach using the Oie *et al.* force field^23^ and DL_POLY^24^,^25^ software, which have both long been used in our group with excellent results.^26^ The use of an atomistic (rather than quantum) approach has two main advantages: (a) the simulations can be extended to a relatively long time (8 ns), which means that a statistically significant number of conformations was sampled, and (b) the solvent molecules can be included in the simulations. In this case, solvent molecules were not included in a continuum model, but using a more accurate explicit model where a simulation box containing 165 solvent molecules (dichloromethane) per each TPA-Ch molecule were taken into account.

## Conclusion

The present study has proven the influence of σ–π orbital interactions on the photophysical and photochemical properties of a chromophore. The concept has been investigated by means of intermolecular TTET, using benzoylthiophene-derived donor moieties (TPA or SUP) covalently linked to a rigid cholesterol scaffold. Photophysical studies (laser flash photolysis and phosphorescence) reveal that fine tuning of the donor triplet energy significantly modifies the rate constants of TTET to appropriate energy acceptors. The experimental results are rationalized by means of theoretical calculations using first principles methods based on DFT as well as molecular dynamics. This principle should be applicable to a wide variety of chromophores, and the concept could be extended to related processes, such as photoinduced electron transfer.

## Supplementary Material

Supplementary information
