# Supplementary material for: Steric shielding vs. σ–π orbital interactions in triplet–triplet energy transfer
Source: Chem Sci. 2015 May 1;6(7):4035–41. doi: 10.1039/c5sc00823a (PMC5707473; doi:10.1039/c5sc00823a)
Supplement: Supplementary file 1 [file SC-006-C5SC00823A-s001.pdf]

Electronic Supplementary Material (ESI) for Chemical Science.
This journal is © The Royal Society of Chemistry 2015

ELECTRONIC SUPPLEMENTARY INFORMATION

Steric shielding *vs* σ–π orbital interactions in triplet-triplet energy transfer

Inmaculada Andreu,[a] Isabel Morera,[b] Fabrizio Palumbo,[c] Germán Sastre,[c] Francisco Bosca,[c, *] and Miguel A. Miranda[c, *]

Table of Contents

1. ^{1}H and ^{13}C-NMR spectra of **(*S*)-SUP-α-Ch** S2

2. ^{1}H and ^{13}C-NMR spectra of **(*R*)-SUP-α-Ch** S3

3. ^{1}H and ^{13}C-NMR spectra of **SUP-β-Ch** S4

4. ^{1}H and ^{13}C-NMR spectra of **TPA- α-Ch_H** S5

5. Optimized geometries using PBE-D3 S6

6. Optimized geometries using M062X-D3 S7

7. Optimized geometries using wB97XD S8

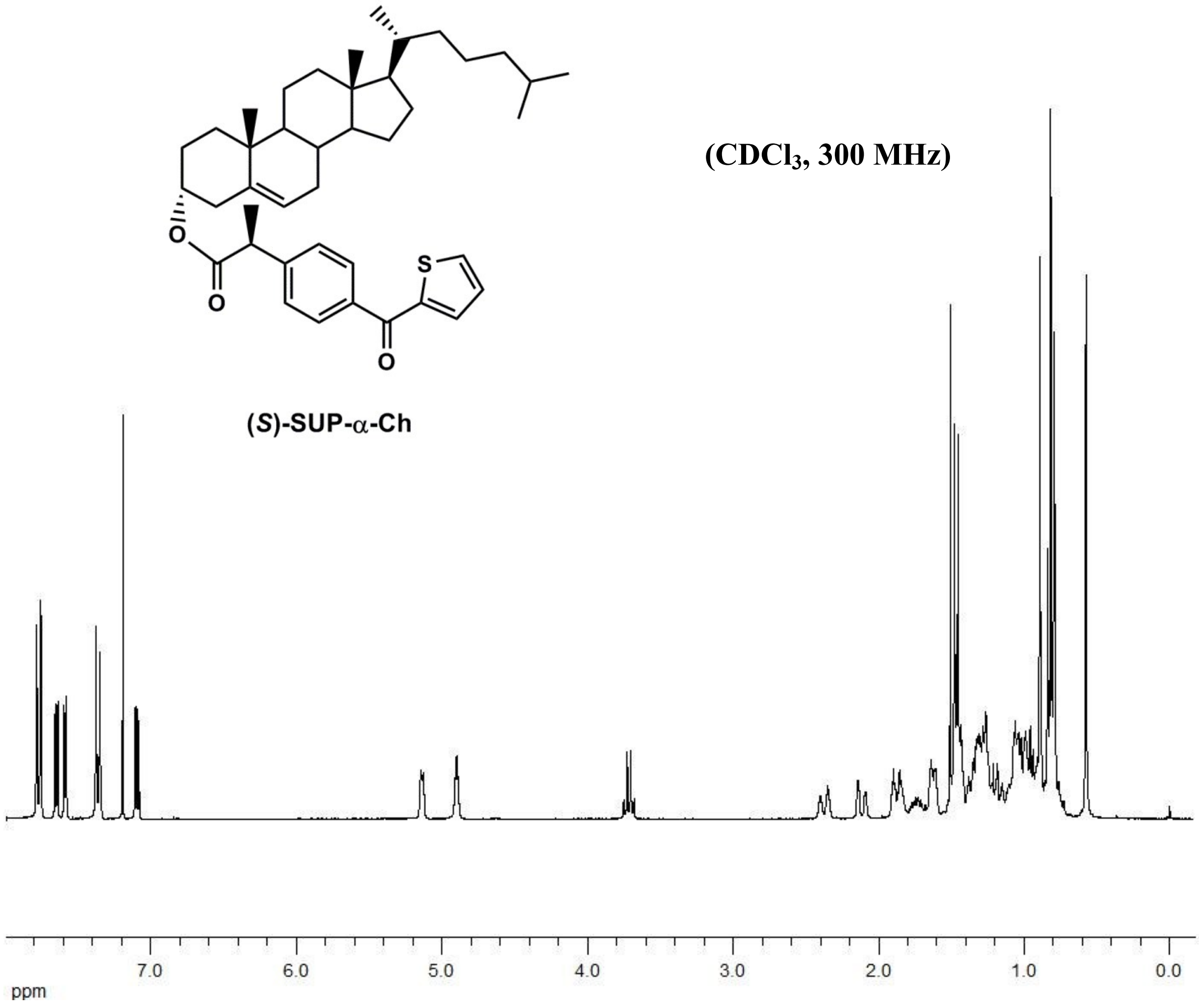
(CDCl3, 300 MHz)
(S)-SUP-α-Ch
7.0
6.0
5.0
4.0
3.0
2.0
1.0
0.0
ppm

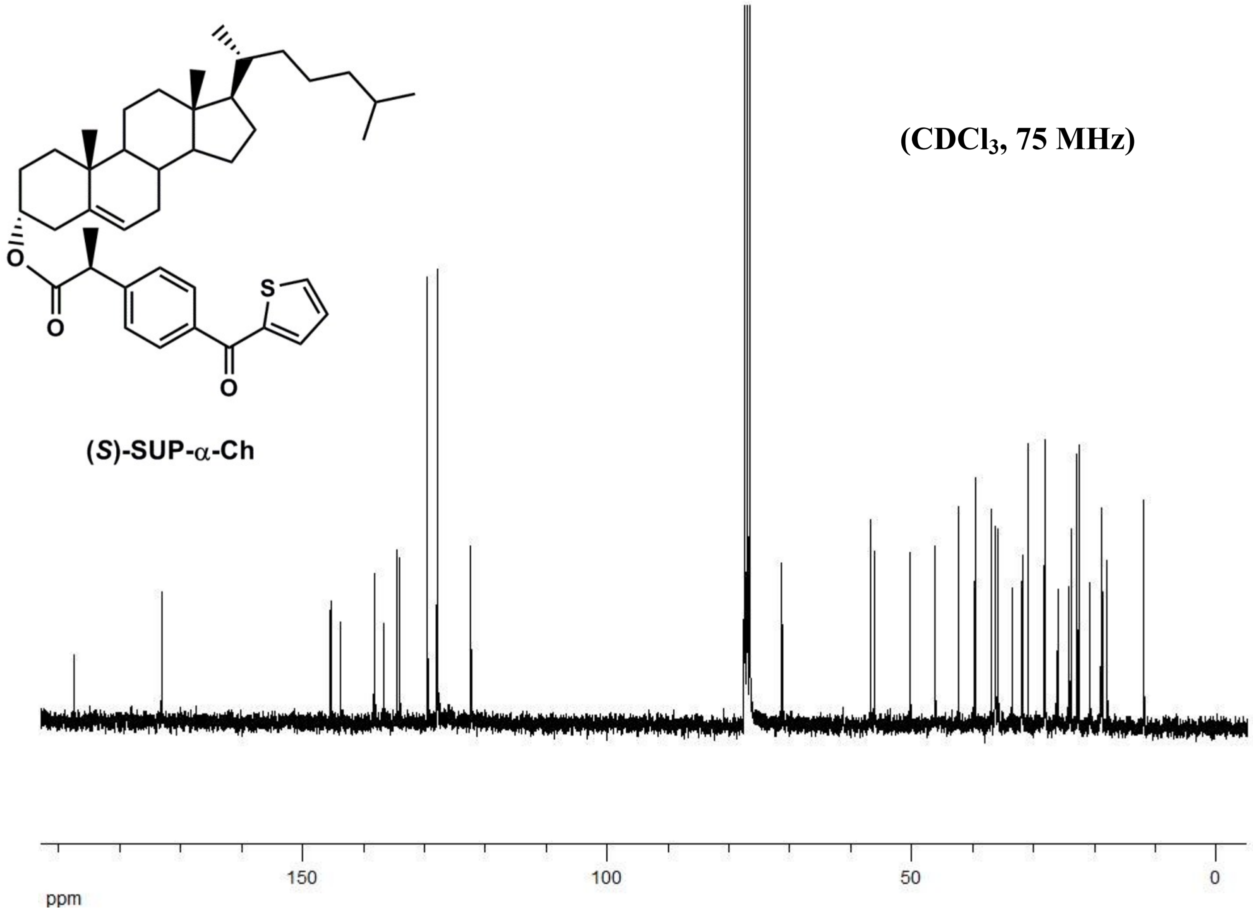
(CDCl3, 75 MHz)
(S)-SUP-α-Ch
150
100
50
0
ppm

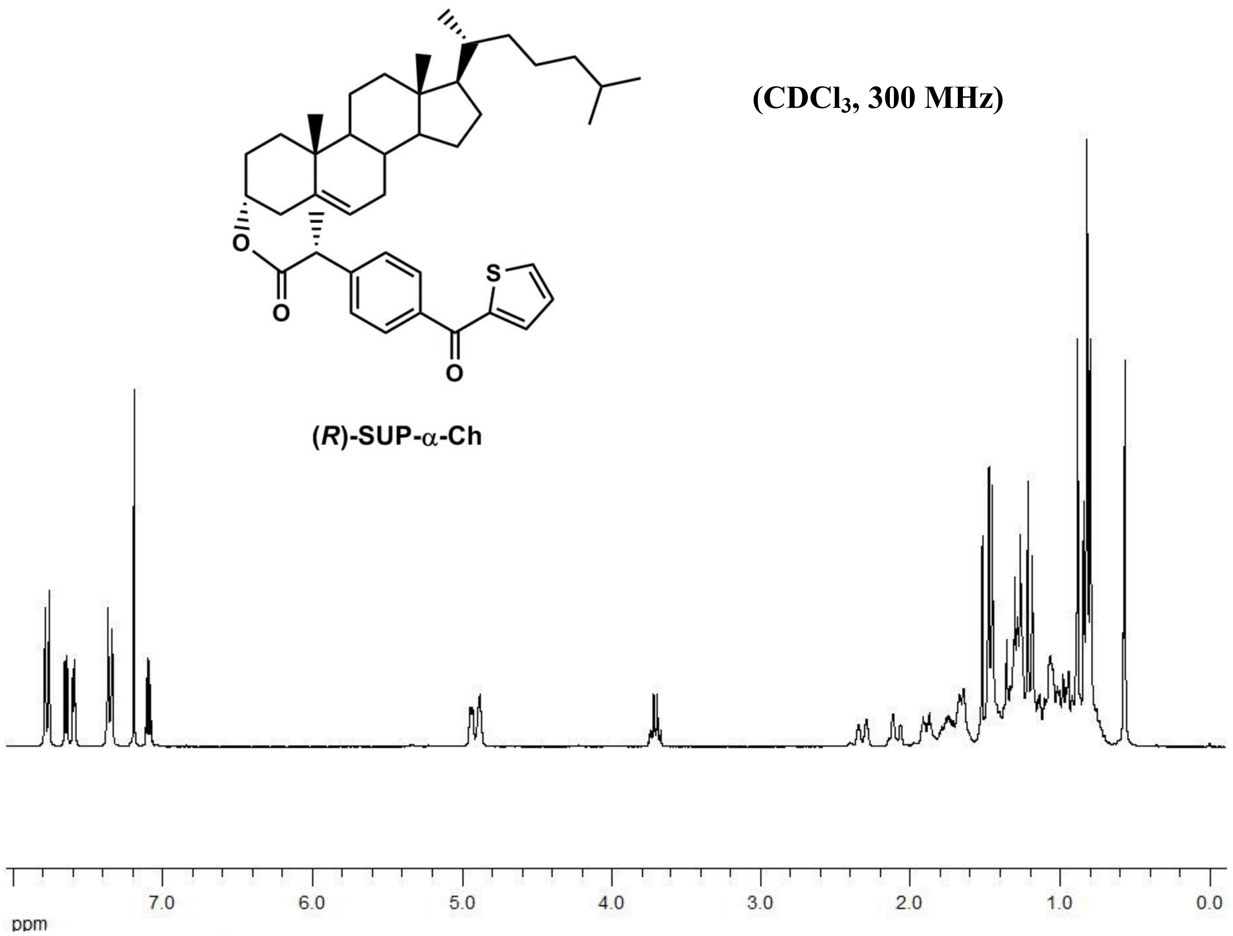
(CDCl$_3$, 300 MHz)
(*R*)-SUP-α-Ch
7.0
6.0
5.0
4.0
3.0
2.0
1.0
0.0
ppm

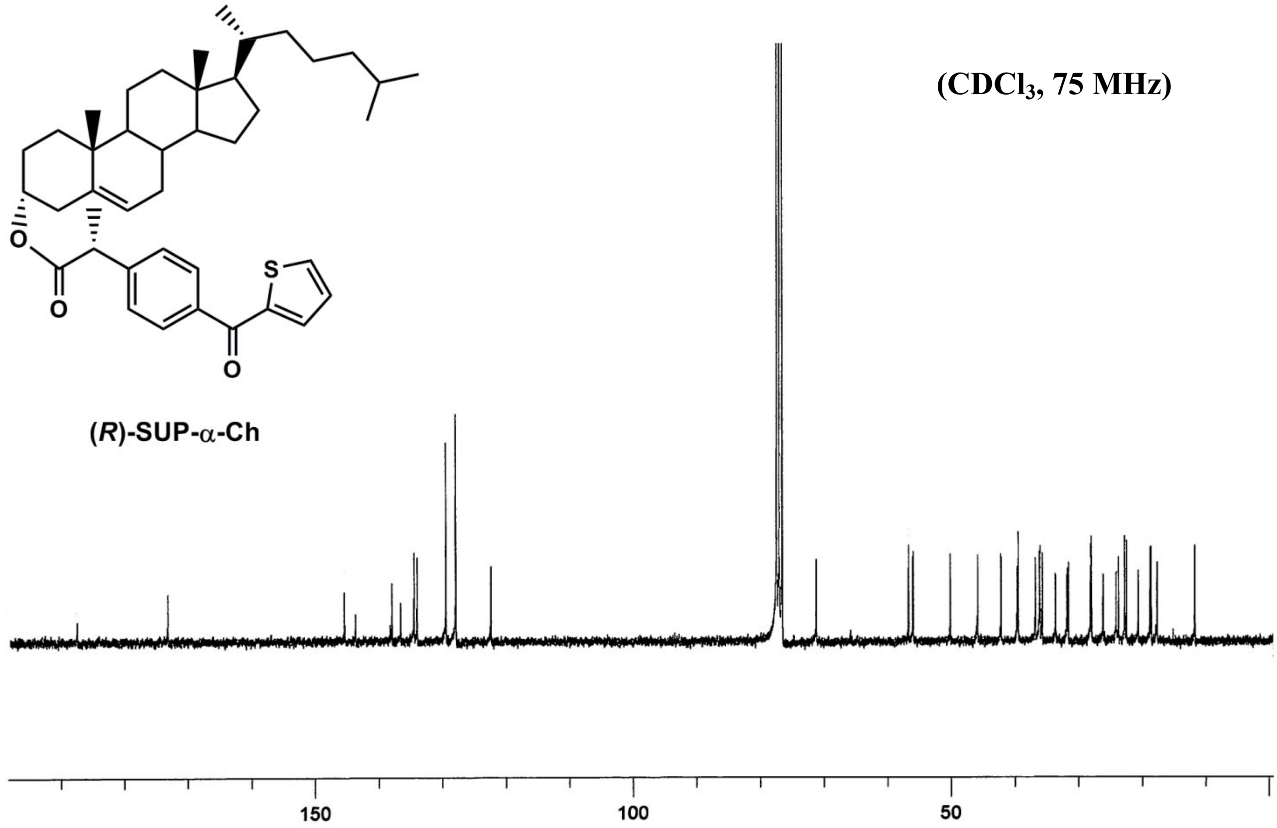
(CDCl$_3$, 75 MHz)
(*R*)-SUP-α-Ch
150
100
50
ppm

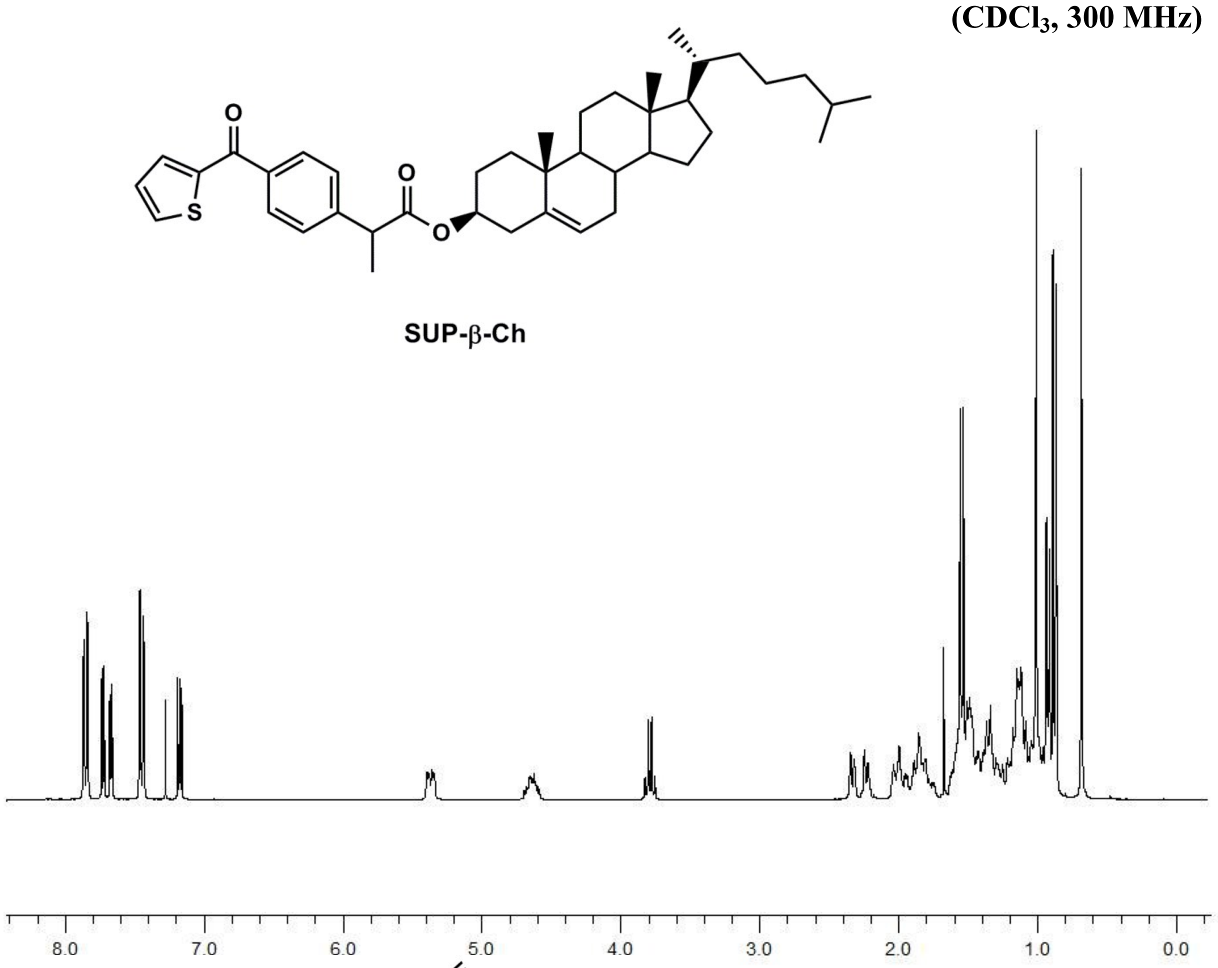
(CDCl3, 300 MHz)
O
O
S
O
SUP-β-Ch
8.0
7.0
6.0
5.0
4.0
3.0
2.0
1.0
0.0

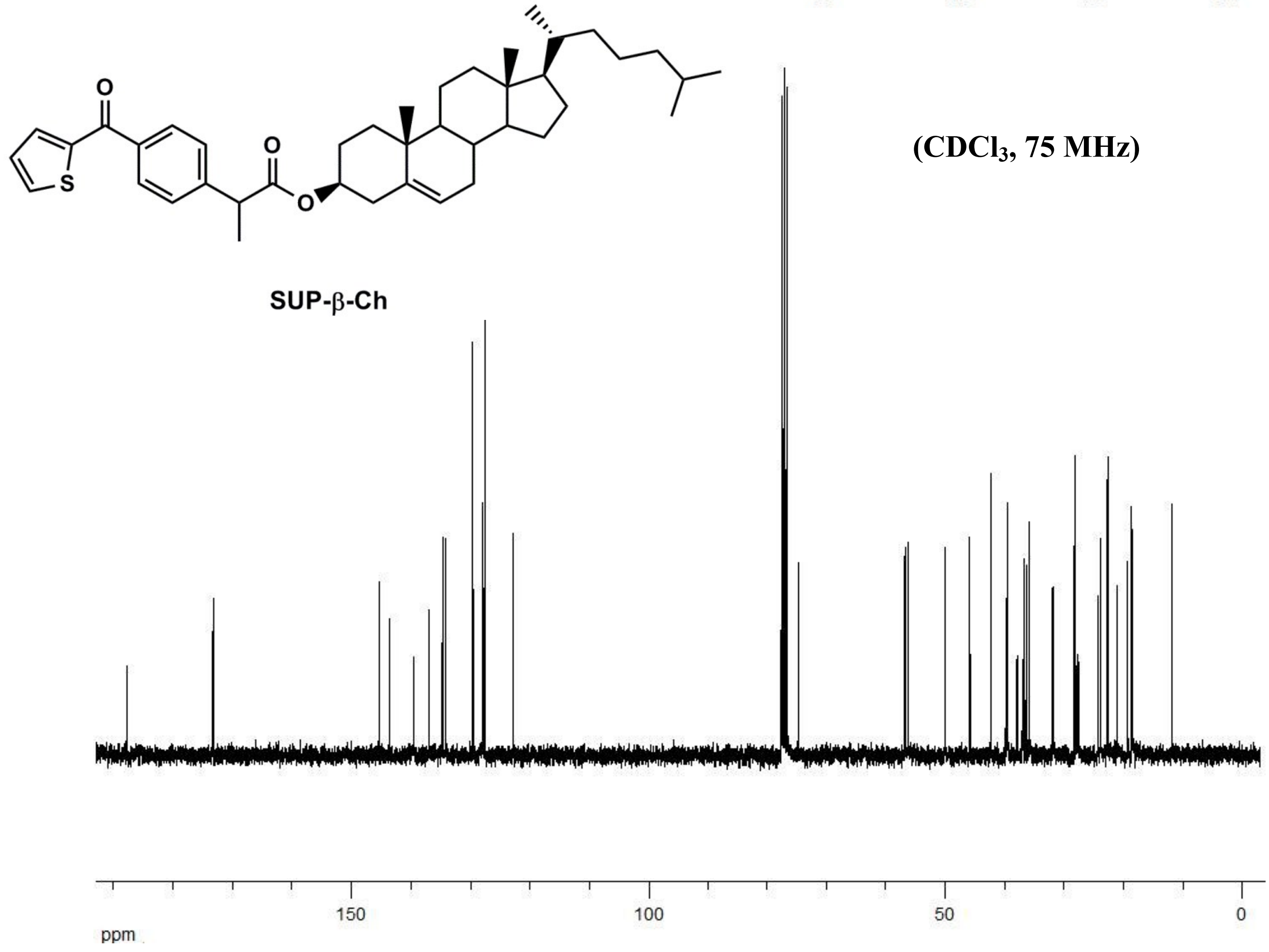
(CDCl3, 75 MHz)
O
O
S
O
SUP-β-Ch
150
100
50
0
ppm

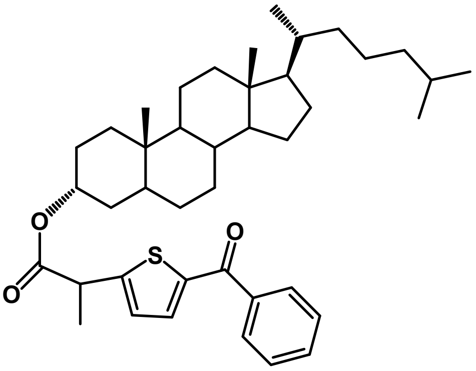

TPA-α-Ch$_H$

(CDCl$_3$, 300 MHz)

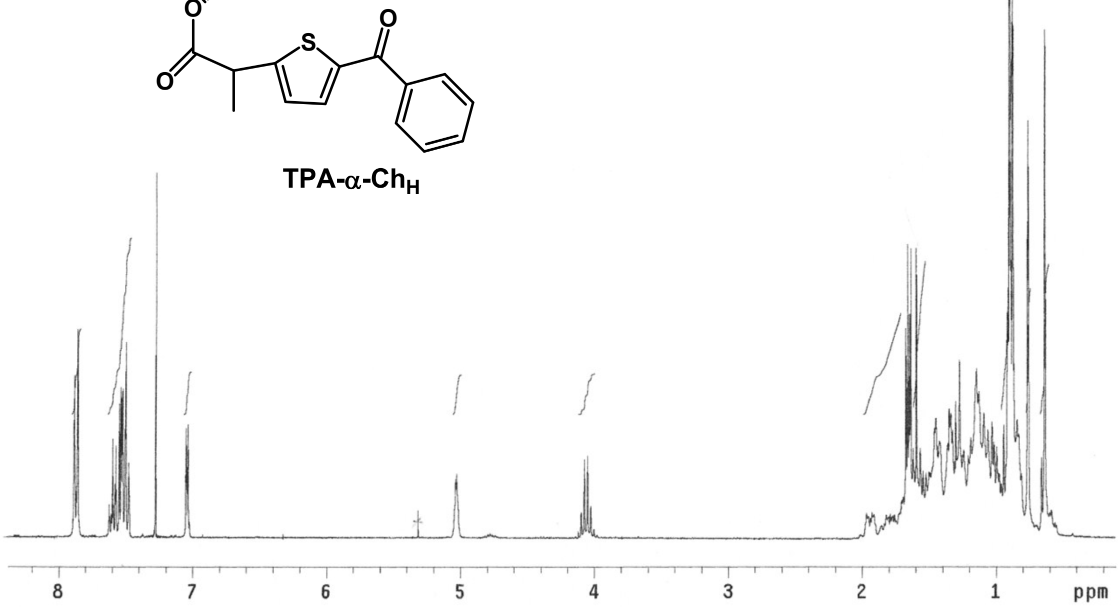

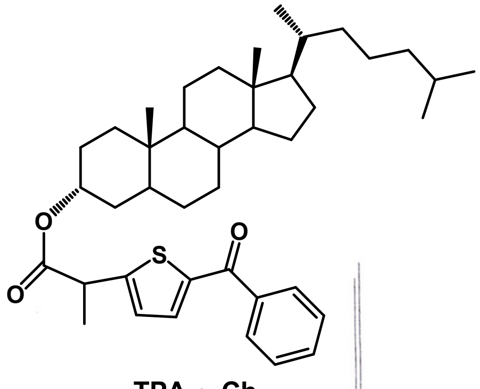

TPA-α-Ch$_H$

(CDCl$_3$, 75 MHz)

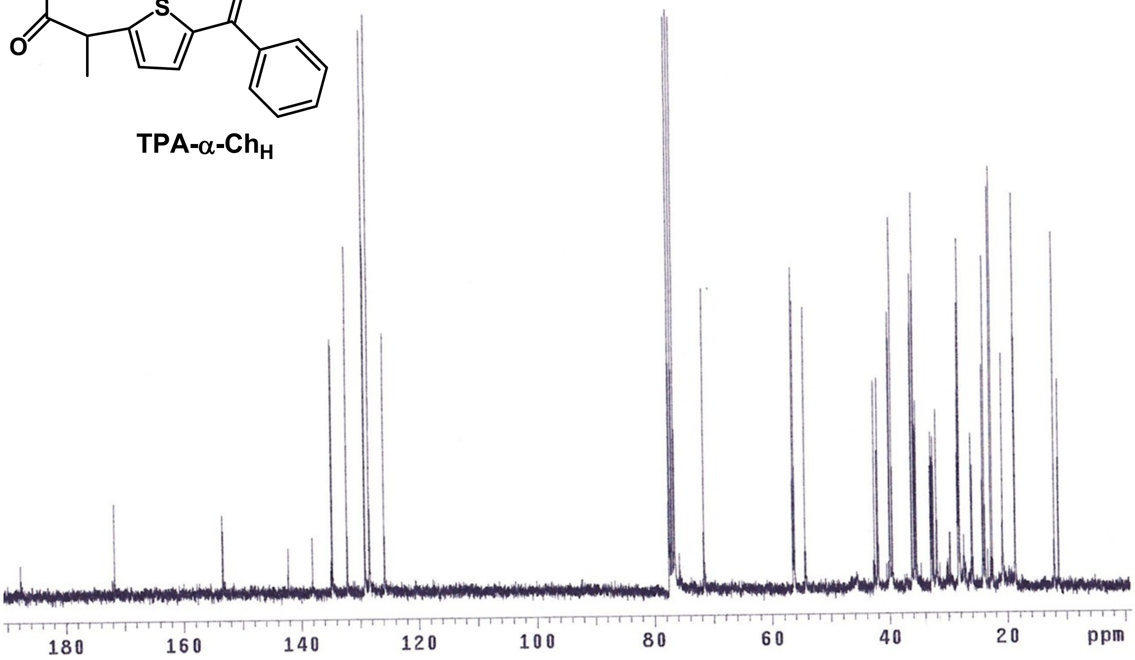

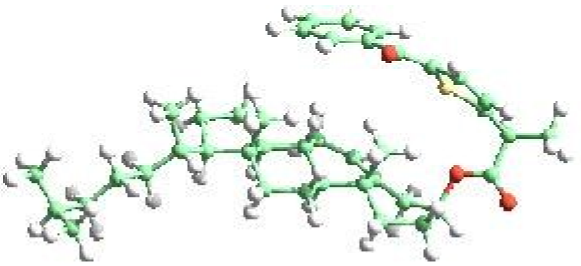

TPA-β-Ch (T_1)

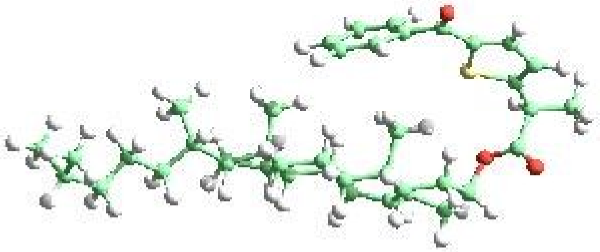

TPA-β-Ch (S_0)

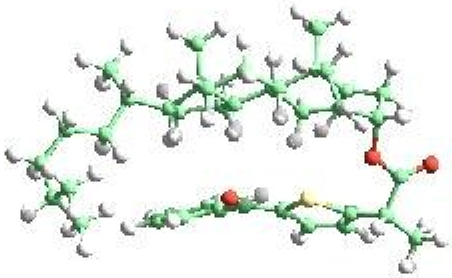

TPA-α-Ch (T_1)

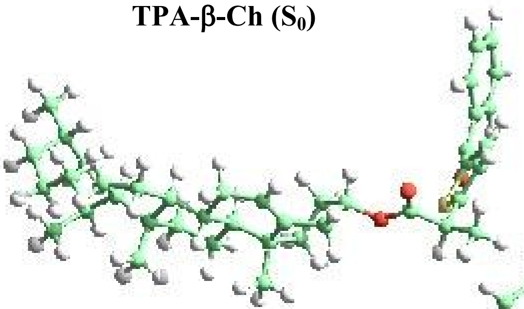

TPA-β-Ch (T_1)

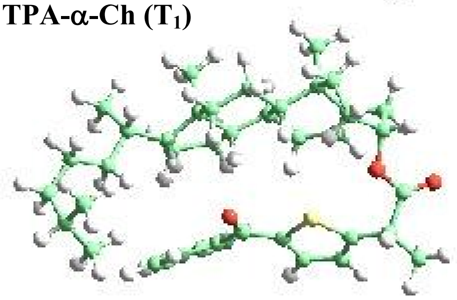

TPA-α-Ch (S_0)

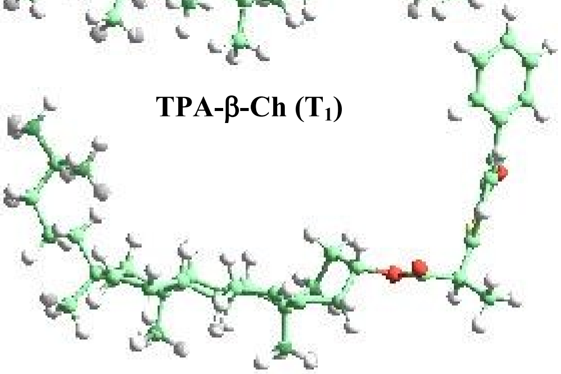

TPA-β-Ch (S_0)

Optimized geometries of ground state (S_0) and triplet excited state (T_1) of TPA-α-Ch and TPA-β-Ch using PBE-D3 functional. The effect of solvent has not been included, but the geometries have also been optimized using dichloromethane as solvent with very similar results. In TPA-β-Ch, folded and unfolded have similar energies and for this reason both conformations have been calculated.

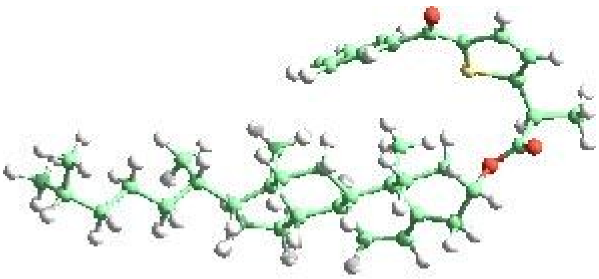

TPA-β-Ch (T_1)

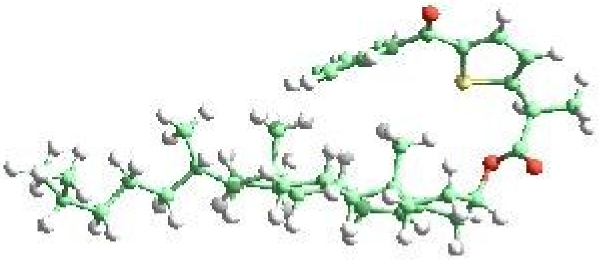

TPA-β-Ch (S_0)

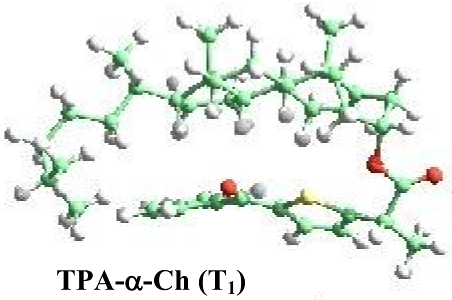

TPA-α-Ch (T_1)

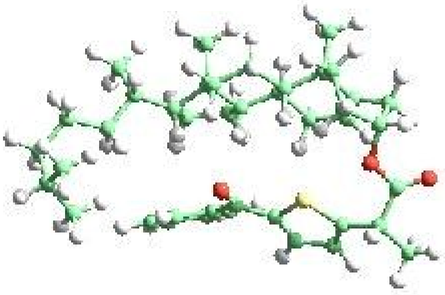

TPA-α-Ch (S_0)

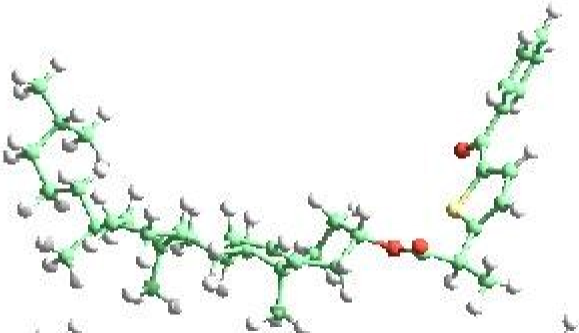

TPA-β-Ch (T_1)

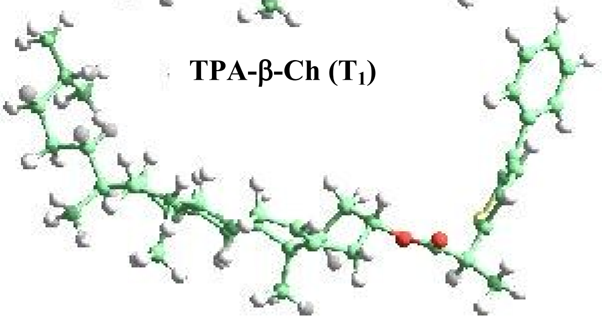

TPA-β-Ch (S_0)

Optimized geometries of ground state (S_0) and triplet excited state (T_1) of TPA-α-Ch and TPA-β-Ch using M062X-D3 functional. The effect of solvent has not been included, but the geometries have also been optimized using dichloromethane as solvent with very similar results. In TPA-β-Ch, folded and unfolded have similar energies and for this reason both conformations have been calculated.

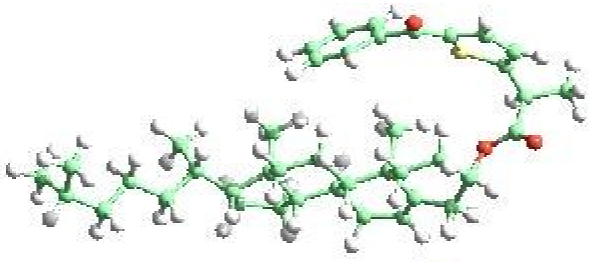

TPA-β-Ch (T_1)

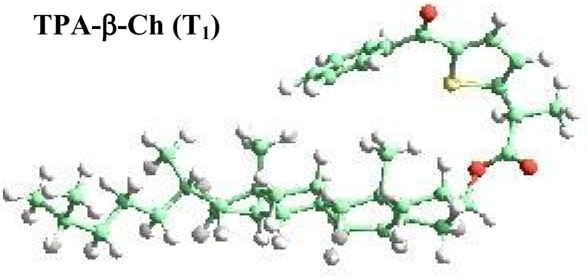

TPA-β-Ch (S_0)

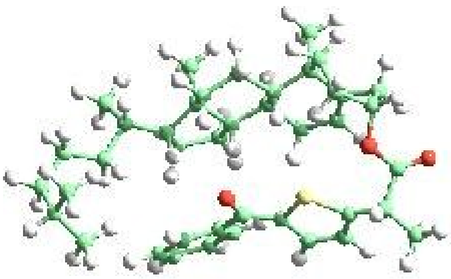

TPA-α-Ch (T_1)

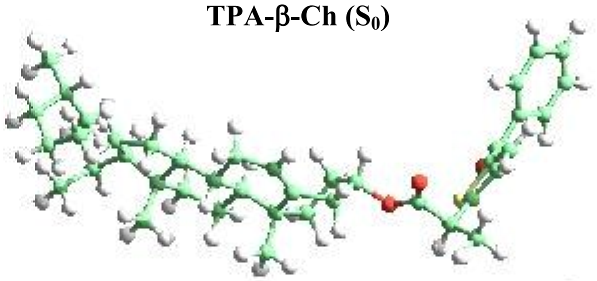

TPA-β-Ch (T_1)

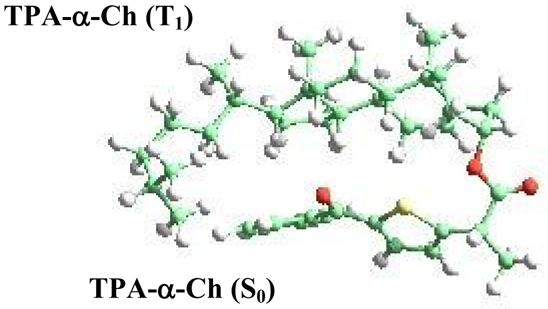

TPA-α-Ch (S_0)

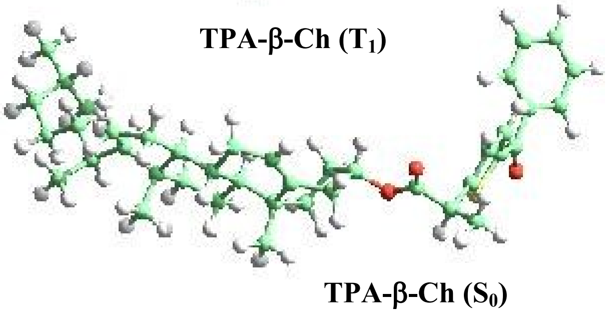

TPA-β-Ch (S_0)

Optimized geometries of ground state (S_0) and triplet excited state (T_1) of TPA-α-Ch and TPA-β-Ch using wB97XD functional. The effect of solvent has not been included, but the geometries have also been optimized using dichloromethane as solvent with very similar results. In TPA-β-Ch, folded and unfolded have similar energies and for this reason both conformations have been calculated.
